# The Metaverse: A New Challenge for the Healthcare System: A Scoping Review

**DOI:** 10.3390/jfmk7030063

**Published:** 2022-08-30

**Authors:** Luca Petrigna, Giuseppe Musumeci

**Affiliations:** 1 Department of Biomedical and Biotechnological Sciences, Section of Anatomy, Histology and Movement Science, School of Medicine, University of Catania, Via S. Sofia n°97, 95123 Catania, Italy; 2 Research Center on Motor Activities (CRAM), University of Catania, Via S. Sofia n°97, 95123 Catania, Italy

**Keywords:** virtual reality, augmented reality, lifelogging, mirror world, health

## Abstract

(1) Background: The metaverse is now a reality, and it interests the scientific community, the educational setting, and medical care. Considering the number of people in front of screens, especially children and adolescents, the metaverse could and should become a place of health promotion. Consequently, the objective of the present study was to review the current literature to detect articles that connected the metaverse with prevention and treatment, education and training, and research setting. (2) Methods: Articles were searched on Pubmed, Web of Science, and Scopus, including English-written papers published until 12 August 2022. They were screened against the eligibility criteria and discussed narratively. (3) Results: The literature published is poor; only 21 articles were included, and 11 of them were added in a second moment. These articles were mainly reviews of the literature or editorials. The aspects related to this virtual world in terms of health prevention and the treatment of clinical conditions, education and training, and research have been narratively discussed. (4) Conclusions: The metaverse could be considered a useful instrument to arrive easily and quickly to the population. Given its importance, today, different studies and investments are required to develop proper health promotion programs that are feasible and valid in the metaverse.

## 1. Introduction

The term “metaverse” was introduced for the first time by Neal Stephenson in 1992, and the science fiction novel Snow Crash is about an immersive and alternative virtual reality, and the internet-connected universe [1] becomes a reality. The metaverse is an internet-based 3-dimensional (3D) virtual world where people conduct daily activities using avatars representing their “real” or imaginary themselves [2]. In a few words, a virtual space became the real world for an alternative life where avatars or digital profiles participate in social activities and in virtual cultural events but also have an economic life [2].

About the structure and technologies behind the metaverse, it can be divided into four different categories: augmented reality, lifelogging, mirror world, and virtual reality [2]:

- Augmented reality adds, in real-time, a digital graphic environment to an existing, physical, and real world. It uses glasses, lenses, or smartphones. In the metaverse, the idea is to superimpose further information on the real environment. Examples are Pokemon Go and 3D medical animations.
- Lifelogging is also an augmentation of the inner word. Different from augmented reality, smart devices are adopted to record daily lives on the internet. Examples are Instagram, Facebook, Twitter, and health monitors.
- A mirror world is a simulation of the real world. The real appearance, information, and structure are transferred to a virtual space, allowing the performance of activities through the internet or mobile applications. Examples are Google Maps or Earth, educational spaces, such as “digital laboratories” and “virtual educational spaces,” but also Zoom, Webex, Google Meet, and Teams.
- Virtual reality is a virtual online sophisticated 3D reality with avatars and an instant communication tool that simulates the inner world. The avatar can be personalized, and the cultural, physical, and social characteristics are different from reality. The avatar can communicate with other entities and achieves goals. Examples are online multiplayer video games, virtual hospitals, and consultation rooms.

The metaverse, therefore, can be considered a location in which the real world is augmented, connected, and replicated with virtual reality and, consequently, as another world [2]. It is also important to consider that for the digital native generation, the metaverse is and will be a space where they spend part of their daily life [2]. These are aspects that have to be considered, especially after the coronavirus disease of the 2019 pandemic that accelerated and implemented the evolution of the metaverse [2,3]. The pandemic situation limited real physical life [4]; consequently, activities such as education, medical care, fashion, shopping, performances, exhibitions, and tourism moved from only offline to be also virtual [2]. So, the metaverse is not only a place to get away from the busy life and enjoy the leisurely life [2], but according to us, it is a place where people will live part of their life using services and facilities. This connection with the metaverse is facilitated by new technologies that allow us to be part of this online world 24 h a day and everywhere. These devices are smartphones and smartwatches, with the last allowing healthcare education, physical activity monitoring, but also the possibility of the self-management of chronic diseases and nursing or home-based care [5].

Diverse industries, private companies, and organizations, from social communications to fashion, from high-tech to business, and from art to real estate, are actively investing and creating virtual entities in the metaverse. The healthcare sector is now starting to deal with the metaverse, and the potentiality of this virtual world for the prevention and treatment of clinical conditions, education and training, and research is limitless. For these reasons and the objective of the present study was to understand the progress of the scientific literature on the metaverse in the healthcare sector and eventually to propose some feedback in terms of health prevention and the treatment of clinical conditions, education and training, and research. Furthermore, the practical utility of the metaverse has also been considered. It is, consequently, important and interesting to review the literature published on the metaverse related to public health to have a clear idea of what it has done and what it is required to do.

## 2. Materials and Methods

The manuscript partially followed the preferred reporting for systematic reviews and meta-analyzes for Scoping Reviews (PRISMA-ScR) checklist and explanation [5]. PRISMA checklist is included in the Supplementary Material. The manuscript was not previously recorded on databases such as PROSPERO.

### 2.1. Search Strategy

The studies were collected through a screening of the electronic databases PubMed (NLM), the Web of Sciences, and Scopus, and the manuscripts were collected if published before 12 August 2022. The only keyword adopted was metaverse to include as many articles as possible in the review.

### 2.2. Eligibility Criteria

No eligibility restrictions were adopted for the population investigated, the intervention adopted, the comparison parameters, the outcomes of the studies, and the study design. The only inclusion criteria were related to the topic that had to be on the metaverse and public health. Only original, peer-reviewed, and English-written manuscripts were included independently of the origin country. No letter to the editor, conference papers, proceedings, or abstracts, while other manuscript typologies were included.

### 2.3. Data Sources, Studies Sections, and Data Extraction

Two investigators performed the study collection and screening. The first step of the screening was the removal of duplicate screenings, which were performed by title, abstract, and full text. Furthermore, in a second step, the references of the included studies were screened to include the review of other studies on this topic.

The main results were summarized in a table where information related to the year of publication, journal, study typology, and the main objective were collected. The results were narratively analyzed and discussed.

## 3. Results

**Figure 1:**
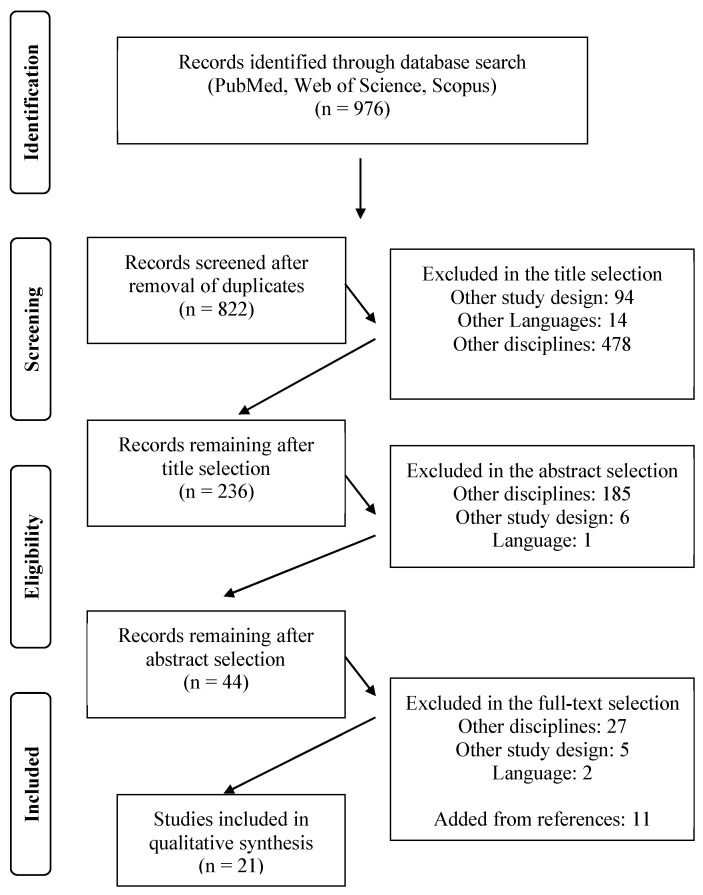
Flow chart of the selection criteria process of the included studies.

A total of 976 (Pubmed: 67; Web of Science: 380; Scopus: 529) studies were detected after the screening of the three databases. Most of the excluded articles were related to other topics. Only 10 articles were included after the eligibility criteria selection. From the references of the included studies, another 11 studies were included. The final number of the included studies was 21. The screening process is presented in Figure 1.

### Characteristics of the Included Studies

Despite no limits being proposed for the year of publication, 1 study was published in 2018 [6] and 1 in 2019 [7]. Five studies were published in 2020, 4 studies in 2021, and 10 studies in 2022. A total of 14 studies were reviewed in the literature, and only one of these studies performed was also a meta-analysis. Five studies were Editorials, one study a prospective, and one study a Global Spotlight. The objectives of the included studies were different, but it was possible to categorize the results in (a) health prevention and the treatment of clinical conditions; (b) education and training, and (c) research. More details are presented in Table 1.

## 4. Discussion

The findings suggest that the literature on this topic is limited to a few reviews of the literature and editorials. The included studies are recent, and the metaverse was adopted with different scopes requiring further investigations in the near future. Despite these limitations, the metaverse can be applied in the health prevention and treatment of clinical conditions; it is feasible in the education and training setting, and researchers can use this tool to make the studies faster and with bigger and worldwide samples. These aspects are deeply presented in the paragraphs below.

### 4.1. Metaverse for Prevention and Treatment

The first aspect that we wanted to analyze is the relation between the metaverse and the prevention and treatment of disease. A bibliometric analysis of virtual reality and augmented reality found that the metaverse can be adopted for diagnostic and surgical procedures and rehabilitation on pain, stroke, anxiety, depression, fear, cancer, and neurodegenerative disorders with satisfying results [23]. Specific, for cancer care, artificial intelligence technologies can be a tool to prevent cancer and diagnose, treat, and rehabilitate patients [21,24].

A widely adopted concept is Health 4.0, and it integrates innovative technologies with health care [4,10]. Examples of Health 4.0 are the Internet of Health Things, medical cyber-physical systems, health cloud or fog, big data analytics, machine learning, blockchain, and smart algorithms [10] but also virtual reality [4]. This allows us to monitor the population but also to educate involving people in community activities. Digital innovations can be adopted as an alternative model of care delivery, and indeed, the possibility to create avatars allows consultations and personalized care [21]. In the metaverse, physicians could visit their patients in a 3D virtual clinic using telemedicine services and home-based devices such as wearable sensors and smartphone applications to monitor their health status [18].

Different devices can be adopted to monitor health conditions directly at home, connecting real life with the virtual world. The monitoring of the clinical aspect and, consequently, the health of the person who is distant can be adopted by 12-lead electrocardiograms for the heart, blood pressure instruments to evaluate the cardiovascular systems, oxygen saturation meters for the cardio-respiratory system, and blood glucose calculators, ideally for people with diabetes [20]. Related to the evaluation of physical performance, also through the web, widely adopted are heart frequency monitors [20]. Another tool widely adopted, especially in the last years, is the smartwatch that integrates heart frequency, blood saturation, pedometers, and accelerometers, but also Global Positioning System (GPS) data allow the monitoring of health status and physical performance, but also the management of chronic disease [5,25,26]. These smartwatches are often connected to the smartphone, and the smartphone is connected to communities where people can compare, in real-time, their data with other users and accept challenges. The potentiality of smartwatches in health promotion programs is huge. Indeed, through real-time monitoring, the possibility of being part of an online community and being guided by experts around the world increases the possibility of attending fitness programs and adopting a healthy lifestyle [27]. These smartwatches could be an instrument to make the person alive in the metaverse. Furthermore, the 24 h a day monitoring of health parameters allows prompt prevention or intervention in case of problems such as atrial fibrillation [28], also helping the service provider to improve the security of the intervention. Along with monitoring, another important aspect to consider is that, in the metaverse, a virtual and artificial intelligence-based avatar or an agent could provide personalized feedback and motivations, and, in this way, the intervention can become more effective in promoting behavior change [29]. These considerations can also be done considering that the avatars, thanks to the new technology, react realistically in their speech, facial expressions, and body language [30]. Virtual reality could be adopted to create pre-operative planning by analyzing the situation in 3D and asking for the opinions of other experts connected through the internet [12,18]. Furthermore, virtual reality can be integrated with different therapies, and it is an effective intervention for various medical conditions with advantages in terms of customization, compliance, cost, accessibility, motivation, and convenience [4]. Virtual reality can also be adopted as a rehabilitation technique, and an example is mirror therapy, which is a way to create a visual illusion that an impaired limb (an example is after a stroke) is correctly working to re-educate the movement of the cognitive patterns [31].

**Figure 2:**
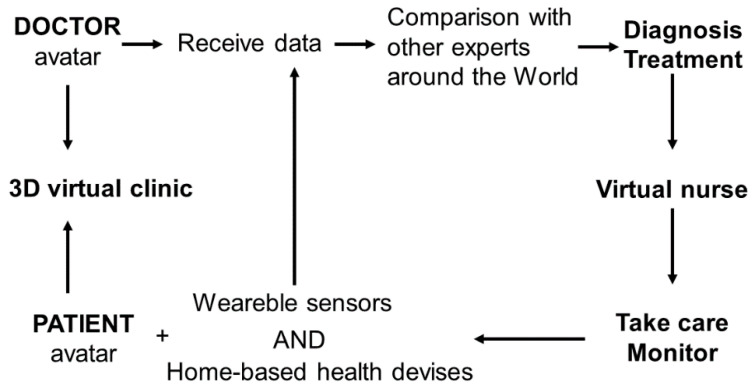
Representation of the possible virtual relationship between doctors and patients.

One last aspect to consider is the possibility of creating an avatar that can act as a “virtual nurse” to direct and monitor care and interact with the patient educating him or the staff around him, but also supervise and monitor, in real-time, the quality/patient safety surveillance, physician activity, and admission and discharge activities [8]. On one side, the metaverse can serve as a transitional stage before real-world experiences [3] with healthcare providers that can accompany patients into specific individualized environments enhancing the efficacy of treatment [3]. On the other side, for chronic diseases such as hypertension, diabetes, obesity, and some mental health conditions, virtual care models with psychological group support programs could be a valid intervention, while remote virtual nursing care with robotic end-user delivery units could also be of help [12]. Regarding mental healthcare, the metaverse with an anonymous virtual realm could be a comfortable place to share stories and other issues with professionals [22]. A representation of this alternative doctor–patient relationship is in Figure 2.

Health promotion can be performed in teaching rooms, and how it is the education of students, the same method can also be adopted for this aspect. However, health promotion programs also mean allowing people to have a space to train. We can imagine virtual rooms in which people can meet with other people and train together. Something similar, just existing and adopted, is the Peloton. It is a virtual environment for bicycling [32]. Peloton is a sports simulator that uses virtual reality, and now, an interactive video where people can compete or exercise together in shared virtual spaces. Users can walk or run on treadmills or by pedaling on stationary bicycles [33]. Obviously, people need exercise equipment that is connected to a local computer through sensors and force feedback controls [33]. In this way, people can train in nature and with other people, inside their houses, offices, or inside the International Space Station where training is a fundamental element [34]. People in their avatar form will be dressed properly, but at home, alone, and they can wear a proper gym suit but also pajamas. The sensors on the equipment will monitor the training of the user and the avatar instructor will guide the users to reach their goals [33]. Feedback could also be the modification of the inclination of the treadmills or the pedaling resistance of their bicycles [33]. People from their homes, offices, or exercise clubs can participate in exercise sessions or compete [33]. According to us, this is an easy way to bring people to move daily, also from their houses, decreasing sedentary behaviors, fundamental aspect nowadays.

### 4.2. Metaverse Education and Training

As the metaverse began to be introduced into present life rapidly, some metaverse applications have already been adopted in education [2], but further improvements are necessary. In an educational setting, virtual reality is the most adopted technology of the metaverse [2,11]. The advantage of virtual reality is that it can be accessed from anywhere, regardless of distance or space [2]. This educational methodology can be adopted by a professor of an important university that can teach people the same methodology in its virtual reality and can also be used for training, for example, its application in surgery, cardiology, and neurology [14]. The overlap of abstract visuals and virtual objects in the context of the real world can be useful for visualizing virtual internal organs and structures of a real body. In this way, it is possible to visualize the internal part of a body directly on the t-shirt of an alive person, or professors could teach aspiring cardiologists the inner workings of the heart and the cardiovascular system in 3D [18]. Some authors used an extended reality technology to create a holographic museum of anatomical structures, such as the eyeball, cerebral venous system, cerebral arterial system, cranial nerves, and various parts of the brain, making it a useful tool in ophthalmic teaching [19]. Like virtual reality, augmented reality can also be adopted to teach during clinical education [11,15]. Both these technologies and virtual rooms allow people to reach each other all around the world and also in remote locations, allowing the standardization of the education of people. This could decrease the discrepancy in the education of future medical students. Virtual reality can be adopted to provide a broad range of training to medical care professionals in anatomy instructions and surgery simulations that can simulate and control different situations that are difficult to duplicate in real life [7].

Another feasible and cheap way to educate people with health education programs organized by experts in the field, universities, or governments is through online platforms. This is a distance learning methodology that can reach people all around the world, 24 h a day, and can be registered while remaining online for an indefinite period of time. Distance learning can also include practical elements such as physical and behavioral education programs.

### 4.3. Metaverse and Research Application

An online system such as the metaverse could provide the possibility of collecting a huge amount of personal health information, allowing the creation of big data and machine learning systems that could help the health care system and research [35]. The data recorded could create national or international monitoring and surveillance systems that researchers could use for their studies, both to obtain data and to compare their results. These digital data could be collected directly from the consumer or from the services or wearables, and they can be shared with a physician, medical professionals, or researchers [18].

**Figure 3:**
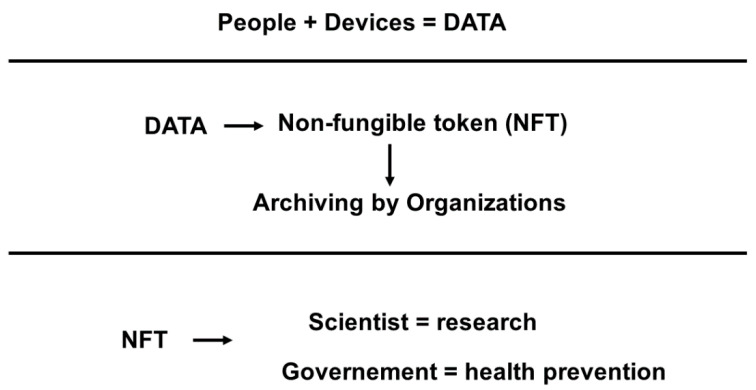
Graphic representation of the passage of the data from people to the researchers and public organizations.

The metaverse is an online world in evolution, and the role of behavioral healthcare providers and researchers in guiding its formation and conducting unbiased research is important [30]. For this reason, it is fundamental to create a solid base of where to start and to try to understand this topic as soon as possible and as well as possible to take the right way. A graphic representation of how the data from people can arrive at researchers and public organizations is in Figure 3.

Another important aspect to consider is the possibility of using extended reality to simulate, within a laboratory, a different location, such as nature or a city, but also a particular setting or a multiple task exercise [36] to stimulate the cognitive system. This kind of technology is just adopted, and examples are glasses or screens. Some manufacturing companies are just selling laboratory instruments, such as the treadmill, associated with screens or special glasses to create an extended reality.

### 4.4. Limit of the Metaverse

The main limits of the metaverse are related to data management and privacy, cybersecurity risks, potential barriers related to access (a lack of internet connectivity), and users with low vision [21]. The metaverse could be a dangerous place with possible new vicious and sophisticated crimes, especially related to stealing personal data [2].

The first aspect to consider is the issues related to the management and storing of data. The commercial interest is huge, and people could have limited power in controlling their data sharing with whom and under what conditions [35]. Consequently, it becomes fundamental to solve the problems related to privacy and security, and technical, legislative, and regulatory problems [20,30]. Also, healthcare leaders have expressed concerns about privacy, ethics, and safety as healthcare moves online [3], making it necessary to enhance data security and centralized regulatory oversight [16]. A method to organize and control access to a complex network of data sets is through blockchain [3,16]. Blockchain is a viable technology that can improve healthcare data sharing and the storing system, owing to its decentralization, immutability, transparency, traceability features, and privacy [3,9]. Blockchain networks integrated with artificial intelligence could help in the personalization of cardiovascular medicine by (a) increasing the data available for developing and training artificial intelligence, (b) sharing algorithms for generalization, and (c) decentralizing databases and incentivizing solutions that improve outcomes [16,17]. Consequently, blockchain could be a new way of encrypting patient data and enforcing compliance with medical standards in practices and processes [20]. The application of blockchain to cardiovascular medicine is still in the beginning, and some concerns about the implementation exist [16,17]. Healthcare organizations are hesitant to adopt blockchain technology due to threats such as security, authorization, and interoperability issues and the lack of technical skills related to blockchain technology [9]. Also, non-fungible tokens could become helpful. Indeed, they allow digital ownership, and they can help incentivize a more democratized, transparent, and efficient system for patients to control their data sharing [35]. In this way, the health system could have the whole medical history, disease, medication, and allergies stored in the patients’ own personalized non-fungible token that only they and their doctor can access (or any other individual that the patient decides to give access to) [20].

The second aspect to consider is the physical relationship. It currently remains much easier to communicate with patients in person, through a phone, or a social media channel than to envision something that seems like a far-fetched science fiction idea [18]. A second limitation is that the metaverse is expected to be actively used in medical and nursing education or education for residents and students [15]. However, even if virtual reality can effectively improve knowledge in nursing education, it is not more effective than other education methods in the areas of skills, satisfaction, confidence, and performance time [13]. Social connections in the metaverse are weaker than interactions in the real world [2]. Furthermore, it is still an expensive technology.

The last aspect is the possible loss of freedom in the future. This is how reality should be, and it is the advantage of the actual metaverse. Consequently, the main issue is if the metaverse must be administrated by the public or under the control of multi-millionaire companies that are developing this alternative reality. Probably, the metaverse should be a representation of the reality with the public and private that will coexist. This means that the government should have to start to invest in this alternative world to guarantee public spaces, educational programs, and public events. One last aspect to consider, both by the public and private administration, is to guarantee personal data privacy with a proper constitution.

The limits of the study are mainly related to the poor quality of the included studies. This suggests that the topic is new and innovative, requiring future investigations. A second limitation of the study is related to the impossibility of having and analyzing real data but only theoretical concepts. Future studies should deeply investigate the feasibility of a health-metaverse in which people can learn and be guided in healthy behaviors. These studies should transform the theoretical concept in original research.

## 5. Conclusions

The metaverse cannot substitute the real world; physical and eye contact, facial expressions, and gestures are essential elements in the healthcare world. However, the metaverse can be considered a tool to improve the quality of the health care system in terms of intervention and treatment, the education of people all over the world, guaranteeing standardized training, and helping the research to create world databases. Finally, considering the time spent by the young population in front of a screen, the metaverse could be a place where they can also start to practice sport and learn something. Sports centers and associations could enrich those citizens who wish to train differently, at home, and when they prefer, increasing the possibilities to be physically active.

## Figures and Tables

**Table 1:** Characteristics of the included studies.

Author	Year	Journal	Study Typology	Main Objective
Abu-Elezz et al. [8]	2020	International Journal of Medical Informatics	Review	Explore and categorise the benefits and threats of blockchain technology application in a healthcare system
All-Jaroodi et al. [9]	2020	IEEE Access	Review	Define Health 4.0 and discuss advanced potential Health 4.0 applications
Almarzouqi et al. [10]	2022	Digital Object Identifier	Review	Evaluate students’ perception of the application of the metaverse for medical-educational purposes
Aziz et al. [6]	2018	Journal of Health & Medical Informatics	Review	Summarizes the current state of knowledge of virtual reality simulation in healthcare
Chapman et al. [11]	2022	SAGE	Editorial	The metaverse is associated with training in lung cancer surgery
Chen et al. [12]	2020	Journal of medical Internet research	Review, meta-analysis	Evaluate the effectiveness of virtual reality in nursing education in the areas of knowledge, skills, satisfaction, confidence, and performance time
Javaid et al. [13]	2020	Clinical Epidemiology and Global Health	Review	Find how the metaverse is going to solve a medical-related problem in saving the life of the patient and what are the significant applications
Kye et al. [2]	2021	Journal of educational evaluation for health professions	Review	Define the 4 types of the metaverse and explain the potential and limitations of its educational applications
Koo et al. [14]	2021	J Educ Eval Health Prof	Editorial	Training in lung cancer surgery through the metaverse
Krittanawong et al. [15]	2021	Canadian Journal of Cardiology	Review	Discuss recent advances and potential future directions for the application of blockchain and its integration with artificial intelligence in cardiovascular medicine
Krittanawong et al. [16]	2020	Nature reviews. Cardiology	Review	Discuss integration of blockchain with artificial intelligencedata-centric analysis and information flow, its limitations, and potential cardiovascular applications
Liu et al. [4]	2022	Int. J. Environ. Res. Public Health	Review	Explore VR in aiding therapy, providing a potential guideline for futures application in healthcare towards Health 4.0
Mesko et al. [17]	2022	European Heart Journal	Global Spotlights	It was associated with the metaverse with cardiovascular health
Ramesh et al. [18]	2022	Indian J Ophthalmol	Editorial	Presentation of the 4D ophthalmic anatomical and pathological metaverse
Schuelke et al. [7]	2019	Nursing administration quarterly	Review	Report on an innovative care system and the effects of this model have on patient satisfaction, patient quality metrics, and financial metrics
Skalidis et al. [19]	2022	Trends in Cardiovascular Medicine	Review	Analysis of the applications of the metaverse and how it can be implemented in cardiovascular medicine
Tan et al. [20]	2022	Asia-Pacific Academy of Ophthalmology	Perspective	Analysis opportunities and challenges of the metaverse in ophthalmology
Usmani et al. [21]	2022	General Psychiatry	Review	Explore the applications of the metaverse on mental health
Wiederhold et al. [3]	2022	Cyberpsychology, behavior, and social networking	Editorial	Application of the metaverse in the health care setting
Yeung et al. [22]	2021	Journal of medical internet research	Review	Association of virtual reality and augmented reality with medicine
Zeng et al. [23]	2022	Asia-Pacific Journal of Oncology Nursing	Editorial	Application of the metaverse in cancer care

## Data Availability

Not applicable.

## Supplementary Materials

The following are available online at https://www.mdpi.com/article/10.3390/jfmk7030063/s1, Figure S1: referred Reporting Items for Systematic reviews and Meta-Analyses extension for Scoping Reviews (PRISMA-ScR) Checklist.

## References

[1] Stephenson N. (1992). Snow Crash: A Novel.

[2] Kye B., Han N., Kim E., Park Y., Jo S. (2021). Educational applications of metaverse: Possibilities and limitations. J. Educ. Eval. Health Prof..

[3] Wiederhold B.K. (2022). Metaverse Games: Game Changer for Healthcare?. Cyberpsychol. Behav. Soc. Netw..

[4] Liu Z., Ren L., Xiao C., Zhang K., Demian P. (2022). Virtual reality aided therapy towards health 4.0: A two-decade bibliometric analysis. Int. J. Environ. Res. Public Health.

[5] King C.E., Sarrafzadeh M. (2018). A survey of smartwatches in remote health monitoring. J. Healthc. Inform. Res..

[6] Tricco A.C., Lillie E., Zarin W., O’Brien K.K., Colquhoun H., Levac D., Moher D., Peters M.D.J., Horsley T., Weeks L. (2018). PRISMA Extension for Scoping Reviews (PRISMA-ScR): Checklist and Explanation. Ann. Intern. Med..

[7] Aziz H.A. (2018). Virtual reality programs applications in healthcare. J. Health Med. Inform..

[8] Schuelke S., Aurit S., Connot N., Denney S. (2019). Virtual nursing: The new reality in quality care. Nurs. Adm. Q..

[9] Abu-Elezz I., Hassan A., Nazeemudeen A., Househ M., Abd-Alrazaq A. (2020). The benefits and threats of blockchain technology in healthcare: A scoping review. Int. J. Med. Inform..

[10] Al-Jaroodi J., Mohamed N., Abukhousa E. (2020). Health 4.0: On the way to realizing the healthcare of the future. IEEE Access.

[11] Almarzouqi A., Aburayya A., Salloum S.A. (2022). Prediction of User’s Intention to Use Metaverse System in Medical Education: A Hybrid SEM-ML Learning Approach. IEEE Access.

[12] Chapman J.R., Wang J.C., Wiechert K. (2022). Into the Spine Metaverse: Reflections on a future Metaspine (Uni-) verse. Glob. Spine J..

[13] Chen F.-Q., Leng Y.-F., Ge J.-F., Wang D.-W., Li C., Chen B., Sun Z.-L. (2020). Effectiveness of virtual reality in nursing education: Meta-analysis. J. Med. Internet Res..

[14] Javaid M., Haleem A. (2020). Virtual reality applications toward medical field. Clin. Epidemiol. Glob. Health.

[15] Koo H. (2021). Training in lung cancer surgery through the metaverse, including extended reality, in the smart operating room of Seoul National University Bundang Hospital, Korea. J. Educ. Eval. Health Prof..

[16] Krittanawong C., Aydar M., Virk H.U.H., Kumar A., Kaplin S., Guimaraes L., Wang Z., Halperin J.L. (2021). Artificial Intelligence–Powered Blockchains for Cardiovascular Medicine. Can. J. Cardiol..

[17] Krittanawong C., Rogers A., Aydar M., Choi E., Johnson K., Wang Z., Narayan S.M. (2020). Integrating blockchain technology with artificial intelligence for cardiovascular medicine. Nat. Reviews. Cardiol..

[18] Mesko B. (2022). The promise of the metaverse in cardiovascular health. Eur. Heart J..

[19] Ramesh P.V., Joshua T., Ray P., Devadas A.K., Raj P.M., Ramesh S.V., Ramesh M.K., Rajasekaran R. (2022). Holographic elysium of a 4D ophthalmic anatomical and pathological metaverse with extended reality/mixed reality. Indian J. Ophthalmol..

[20] Skalidis I., Muller O., Fournier S. (2022). CardioVerse: The Cardiovascular Medicine in the Era of Metaverse. Trends Cardiovasc. Med..

[21] Tan T.F., Li Y., Lim J.S., Gunasekeran D.V., Teo Z.L., Ng W.Y., Ting D.S. (2022). Metaverse and Virtual Health Care in Ophthalmology: Opportunities and Challenges. Asia-Pac. J. Ophthalmol. (Phila. Pa.).

[22] Usmani S.S., Sharath M., Mehendale M. (2022). Future of mental health in the metaverse. Gen. Psychiatry.

[23] Yeung A.W.K., Tosevska A., Klager E., Eibensteiner F., Laxar D., Stoyanov J., Glisic M., Zeiner S., Kulnik S.T., Crutzen R. (2021). Virtual and augmented reality applications in medicine: Analysis of the scientific literature. J. Med. Internet Res..

[24] Zeng Y., Zeng L., Zhang C., Cheng A.S. (2022). The Metaverse in Cancer Care: Applications and Challenges.

[25] Jat A.S., Grønli T.-M. Smart Watch for Smart Health Monitoring: A Literature Review. Proceedings of the International Work-Conference on Bioinformatics and Biomedical Engineering.

[26] Debon R., Coleone J.D., Bellei E.A., De Marchi A.C.B. (2019). Mobile health applications for chronic diseases: A systematic review of features for lifestyle improvement. Diabetes Metab. Syndr. Clin. Res. Rev..

[27] Amagai S., Pila S., Kaat A.J., Nowinski C.J., Gershon R.C. (2022). Challenges in participant engagement and retention using mobile health apps: Literature review. J. Med. Internet Res..

[28] Isakadze N., Martin S.S. (2020). How useful is the smartwatch ECG?. Trends Cardiovasc. Med..

[29] Taylor L., Ranaldi H., Amirova A., Zhang L., Ahmed A.A., Dibb B. (2022). Using virtual representations in mHealth application interventions for health-related behaviour change: A systematic review. Cogent Psychol..

[30] Wiederhold B.K. (2022). Ready (or Not) player one: Initial musings on the metaverse. Cyberpsychol. Behav. Soc. Netw..

[31] Zhang Y., Xing Y., Li C., Hua Y., Hu J., Wang Y., Ya R., Meng Q., Bai Y. (2022). Mirror therapy for unilateral neglect after stroke: A systematic review. Eur. J. Neurol..

[32] Ensor J.R., Carraro G.U. (1998). Peloton: A distributed simulation for the world wide web. Simul. Ser..

[33] Carraro G.U., Cortes M., Edmark J.T., Ensor J.R. The peloton bicycling simulator. Proceedings of the Third Symposium on Virtual Reality Modeling Language.

[34] Petrigna L., Bianco A. (2022). Is the human body able to travel on Mars?. Sci. Sports.

[35] Kostick-Quenet K., Mandl K.D., Minssen T., Cohen I.G., Gasser U., Kohane I., McGuire A.L. (2022). How NFTs could transform health information exchange. Science.

[36] Petrigna L., Gentile A., Mani D., Pajaujiene S., Zanotto T., Thomas E., Paoli A., Palma A., Bianco A. (2021). Dual-Task Conditions on Static Postural Control in Older Adults: A Systematic Review and Meta-Analysis. J. Aging Phys. Act..

